# Prevalence of latent tuberculosis and associated factors in patients with chronic kidney disease on hemodialysis

**DOI:** 10.1590/1518-8345.3839.3442

**Published:** 2021-07-19

**Authors:** Viviane Ferreira, Cassiane Dezoti da Fonseca, Valdes Roberto Bollela, Elen Almeida Romão, Jose Abrão Cardeal da Costa, Alvaro Francisco Lopes de Sousa, Dulce Aparecida Barbosa

**Affiliations:** 1 Universidade Federal de São Paulo, Escola Paulista de Enfermagem, São Paulo, SP, Brazil.; 2 Universidade de Araraquara, Araraquara, SP, Brazil.; 3 Centro Universitário Estácio Ribeirão Preto, Ribeirão Preto, SP, Brazil.; 4 Universidade de São Paulo, Faculdade de Medicina de Ribeirão Preto, Departamento de Clínica Médica, Divisão de Moléstias Infecciosas e Tropicais, Ribeirão Preto, SP, Brazil.; 5 Universidade de São Paulo, Faculdade de Medicina de Ribeirão Preto, Departamento de Clínica Médica, Divisão de Nefrologia, Ribeirão Preto, SP, Brazil.; 6 Universidade Nova de Lisboa, Instituto de Higiene e Medicina Tropical, Lisboa, Portugal.; 7 Scholarship holder at the Conselho Nacional de Desenvolvimento Científico e Tecnológico (CNPq), Brazil.

**Keywords:** Chronic Kidney Diseases, Diabetes *Mellitus*, Hemodialysis, Tuberculin Test, Renal Replacement Therapy, Latent Tuberculosis, Doença Renal Crônica, Diabetes *Mellitus*, Mellitus, Hemodiálise, Teste Tuberculínico, Terapia de Substituição Renal, Tuberculose Latente, Enfermedad Renal Crónica, Diabetes *Mellitus*, Mellitus, Hemodiálisis, Prueba de Tuberculina, Terapia de Reemplazo Renal, Tuberculosis Latente

## Abstract

**Objective:**

to identify the prevalence of latent tuberculosis in patients with chronic kidney disease on hemodialysis and associated factors.

**Method:**

a cross-sectional study conducted with 176 patients with chronic kidney disease on hemodialysis. The tuberculin test was performed with the standardized antigen, distributed by the Brazilian Ministry of Health, and the reading occurred after 72 to 96 hours of the application. An association test (Chi-square, Fisher’s exact), prevalence ratio, and multivariate regression tests were performed.

**Results:**

the prevalence of latent tuberculosis diagnosed through Tuberculosis Skin Test was 8.5% (15/176). The “has/has had diabetes” (aOR: 0.117; 95%CI: 0.015-0.92) and “having regular garbage collection (aOR: 0.076; 95%CI: 0.008-0.702) factors were associated with a lower probability of having a Positive skin test.

**Conclusion:**

the low prevalence of latent tuberculosis identified and the factors associated with it reinforce the need for screening for latent tuberculosis infection for diabetics combined with an analysis of previous risk factors and comorbidities.

## Abstract (pt)

**Objetivo:**

identificar a prevalência de tuberculose latente em pacientes com doença renal crônica em hemodiálise e fatores associados.

**Método:**

estudo transversal realizado com 176 pacientes com doença renal crônica em hemodiálise. O teste tuberculínico foi realizado com o antígeno padronizado, distribuído pelo Ministério da Saúde do Brasil, e a leitura ocorreu após 72 a 96 horas da aplicação. Foram realizados teste de associação (Qui-quadrado, exato de Fisher), razão de prevalência e regressão multivariada.

**Resultados:**

a prevalência de tuberculose latente (teste tuberculínico positivo) foi de 8,5% (15/176). Os fatores “tem/teve diabetes” (ORa:0,117; IC95% 0,015-0,92) e “ter coleta de lixo regular” (ORa:0,076; IC95% 0,008-0,702) foram associados a menores probabilidades de ter teste tuberculínico positivo.

**Conclusão:**

a baixa prevalência de tuberculose latente identificada e os fatores associados à mesma reforçam a necessidade de uma triagem da infecção latente por tuberculose para diabéticos combinada com a análise de fatores de risco e comorbidades prévias.

## Abstract (es)

**Objetivo:**

identificar la prevalencia de tuberculosis latente en pacientes con enfermedad renal crónica en hemodiálisis y factores asociados.

**Método:**

estudio transversal realizado con 176 pacientes con enfermedad renal crónica en hemodiálisis. La prueba cutánea de la tuberculina se realizó con el antígeno estandarizado, distribuido por el Ministerio de Salud de Brasil, y la lectura se realizó después de 72 a 96 horas de la aplicación. Se realizaron pruebas de asociación (Chi-cuadrado, exacta de Fisher), razón de prevalencia y regresión multivariante.

**Resultados:**

la prevalencia de tuberculosis latente (prueba de tuberculina positiva) fue de 8,5% (15/176). Los factores “tiene/ha tenido diabetes” (ORa: 0,117; IC del 95%: 0,015-0,92) y “tener recolección regular de residuos” (ORa: 0,076; IC del 95%: 0,008-0,702) se asociaron con menores probabilidades de tener una prueba de tuberculina positiva.

**Conclusión:**

la baja prevalencia de tuberculosis latente registrada y sus factores asociados refuerzan la necesidad del cribado de la infección tuberculosa latente en diabéticos combinado con el análisis de factores de riesgo y comorbilidades previas.

## Introduction

Tuberculosis (TB) is still a severe public health problem worldwide, being among the 10 main causes of death^1^. Official data from the World Health Organization^1^^-^ 2 report that nearly 10 million people in the world have fallen ill with TB every year and that more than 1.5 million have died from the disease in 2018.

Ending the global TB pandemic is one of the goals of the Sustainable Development Goals (SDGs), which aim to reduce by 90% the deaths and by 80% the incidence of the disease until 2030, comparing with the 2015 levels^2^. Brazil still records a high prevalence of TB, with 72,000 new cases in 2018, focused mainly on minorities (prisoners, people with HIV/AIDS, homeless population) more vulnerable to the disease^3^.

Patients with some type of immunosuppression, such as those with Chronic Kidney Disease (CKD), are more susceptible to TB infection^4^. In these patients, uremia reduces the expression of the co-stimulatory B7-2 molecule in cells presenting the antigen, which alters the function of the polymorphonuclear cells and interferes in the phagocytic, migration, and chemotactic efficiency, reducing the capacity of cells to kill intracellular microorganisms. In patients with CKD on hemodialysis, the risk of developing TB is 6.5 to 52.5 times higher than in the general population^4^^-^ 5.

These patients present great risk of developing active TB from a previous latent infection^6^ and greater risk of reactivating latent TB, or even its atypical presentation. It is because of this that there are explicit recommendations for the investigation of latent TB in patients that are being or will be subjected to immunosuppressive therapies/conditions, including CKD and transplanted individuals^7^.

A considerable gap in the knowledge of the prevalence of latent TB and associated factors in patients with CKD on hemodialysis was observed in the literature in general^7^, mainly in Brazil^3^, which makes it difficult to establish a situation overview and thus setting up public policies.

In Brazil, the TB skin test, also called a Mantoux tuberculin skin test (TST), is a classic diagnostic tool to identify latent TB and is essential for patients with CKD on hemodialysis^8^. Considering that the strategies in the identification of chronic kidney patients carriers of the latent form of the Koch’s bacillus are still incipient and the fact of Brazil being an endemic country of this disease in the world, we aim to identify the prevalence of latent tuberculosis in patients with chronic kidney disease on hemodialysis and associated factors.

## Method

This is a cross-sectional study conducted from July to December 2018 in Kidney Replacement Therapy units of the Clinical Hospital (HCFMRP-USP), of the Ribeirão Preto Nephrology Service (SENERP), Northwest of São Paulo, Brazil, which add up to a mean of 11,055 to 45,530 hemodialysis sessions/year and are references in dialysis treatment in the region.

We considered the total population of patients with terminal CKD on hemodialysis kidney replacement therapy, assisted in this period, including patients successively in the research, once they met the inclusion criteria: patients from both gender, aged 18 years old or older. The exclusion criteria were patients with terminal CKD on hemodialysis replacement therapy who presented clinical manifestations and complementary tests suggestive of active TB. For that, the chest x-ray was evaluated and atypical findings were re-evaluated using a chest computed tomography.

After signing the free and informed consent form, an interview was conducted with the patients. Sociodemographic (socioeconomic and health conditions of the patients) and clinical variables were collected by means of a questionnaire, created and validated for this research, taking into consideration vulnerabilities already consolidated in the literature^5^^,^ 8.

The data collection instrument was divided into four sections that covered sociodemographic (gender, age, weight, income, housing, sewage, and garbage collection among others) and clinical variables. The cause of CKD was indicated by the participants, as well as the confirmation of its cause by renal biopsy, the date of diagnosis, type of dialysis, initiation and access. The comorbidities were surveyed by self-report, as well as the time of diagnosis. Data related to the history of TB were asked, and the BCG vaccine was evaluated on the vaccination chart and the scar confirmed by the main researcher.

The TST was performed by a nurse qualified for the procedure under supervision of the researcher, using the Mantoux technique, as follows: it was applied intradermally on the middle third of the anterior face of the left forearm, at a dose of 0.1 ml, which contains 2 TUs (tuberculin units) of the antigen standardized and distributed by the Ministry of Health (PPD RT23, 2 UT-State Serum Institute, Copenhagen, Denmark). The reading occurred between 72 and 96 hours after the application. The measurement was taken with a millimeter ruler, corresponding to the measurement (in mm) of the greatest transverse diameter of the hardening area of the palpable spot^8^. The result was recorded in millimeters, with the following classification and clinical interpretation: 0 mm to 4 mm (no reactor/negative tuberculin) and over 5 mm (reactor/positive tuberculin). Those reactors were duly informed of the result, and the necessary procedures for care and follow-up were carried by the institution itself.

For the univariate statistics and analysis of the association between the kidney chronic patient’s interest variables with the results of the TST, the Chi-square test and Fisher’s exact test were used. For the quantitative variables, analysis of variance (ANOVA) was used. The multivariate analysis was performed by means of Poisson Regression with robust variation. For this multivariate analysis, the outcome (TST, categorized in reactor or non-reactor) was included, as well as each of the independent variable associated with it with a p-value≤0.20, using the stepwise procedure. The insertion sequence of each term in the model was determined by the mutual analysis of the theoretical relevance criteria and statistical significance. Each term was added or removed from the model after identifying statistical significance (p-value<0.05). To build the confidence intervals, a 95% confidence interval was used, with the support of the Statistical Package for the Social Sciences (SPSS) IBM^®^ Version 23.0.

The study was approved by the Research Ethics Committee of the Federal University of São Paulo and of the Clinical Hospital of the Medical School of Ribeirão Preto, University of São Paulo (*Hospital das Clínicas da Faculdade de Medicina de Ribeirão Preto da Universidade de São Paulo*, HCFMRP-USP), under protocol No. 2.489.305. The participating patients signed the free and informed consent form, following Resolution 466/2012 of the National Health Council.

## Results

In the sample of 176 patients, the study estimated a prevalence of 8.5% reactors (positive TST). Table 1 reveals the relationship of the tuberculin test with the patients’ sociodemographic and clinical variables.

**Table 1:** Association between the studied variables with the results of the Tuberculin skin tests in the chronic kidney patients, Ribeirão Preto, SP, Brazil, 2018

Variables		Tuberculin skin test (TST) Results		PR^*^ (95% CI)	p^†^
		Non Reactor n (%)	Reactor n (%)		
Gender					
	Female	67 (41.6)	5 (33.3)	Ref.	0.53
	Male	94 (58.4)	10 (66.7)	1.38 (0.49- 3.88)	
Skin color					
	White	99 (61.5)	8 (53.3)	Ref.	0.55
	Brown	25 (15.5)	4 (26.7)	0.54 (0.18- 1.67)	
	Black	37 (23.0)	3 (20.0)	1.00 (0.28 - 10.89)	
Systemic Arterial Hypertension					
	Absent	110 (68.4)	1 (6.7)	-	0.20
	Present	51 (31.6)	14 (93.3)	3.6 (0.49- 26.48)	
*Diabetes Mellitus*					
	Absent	101 (62.7)	14 (93.3)	-	0.02
	Present	60 (37.3)	1 (6.7)	0.13 (0.02- 0.99)	
Garbage collection					
	No	2 (1.2)	2 (13.3)	Ref	0.04
	Yes	159 (98.8)	13 (86.7)	0.15 (0.05 - 0.46)	
BCG Vaccine					
	No	21 (13.0)	1 (6.7)	Ref	0.41
	Yes	140 (87.0)	14 (93.3)	2.00 (0.28 - 14.47)	
BCG Scar					
	No	35 (24.7)	4 (26.7)	Ref	0.75
	Yes	126 (78.3)	11 (73.3)	0.78 (0.26 - 2.32)	
Previous Tuberculin skin test (TST)					
	No	128 (79.5)	10 (66.7)	Ref	0.32
	Yes	33 (20.5)	5 (33.3)	1.82 (0.66 -5.21)	
Resident in the house had tuberculosis					
	No	144 (89.4)	13 (86.7)	Ref	1.00
	Yes	17 (10.6)	2 (13.3)	1.27 (0.31 -5.21)	
Contact with TB individuals					
	No	156 (96.9)	14 (93.3)	Ref	0.42
	Yes	5 (3.1)	1 (6.7)	2.02 (0.32 - 12.98)	
HIV Test					
	Negative	158 (98.1)	15 (100)	-	1.00
	Positive	3 (1.9)	0 (0.0)	-	
Total		161 (91,4)	15 (8,6)	-	-

* PR = Prevalence Ratio;

† p = p-value

The multivariate analysis (Table 2) indicated the probability of having a reactor or a positive TST. The patient who has/has had diabetes is 8% less likely to be a positive TST factor (PR:0.117; 95%CI: 0.015-0.92). On the other hand, having regular garbage collection shows a 92% lower chance of being positive TST factor (PR:0.076; 95%CI: 0.008-0.702).

**Table 2:** Multivariate binary logistic regression model of factors associated with the tuberculosis skin test results in the chronic kidney patients, Ribeirão Preto, SP, Brazil, 2018

Variable	p^*^	PR	95% CI^†^	
			Lower	Upper
Garbage Collection	0.023	0.076	0.008	0.702
*Diabetes Mellitus*	0.040	0.117	0.015	0.92

* p = p-value;

† CI = Confidence interval

## Discussion

We recorded a low prevalence of latent TB among patients with CKD on hemodialysis (8.5%), when compared to other international and national surveys. A study conducted in Taiwan^9^ reported a prevalence of 11%, very close to another study carried out in Brazil^5^, which recorded a prevalence of 11.2% in the same conditions. Among the predictive factors for latent TB, we highlight the presence of pre-existing comorbidities (diabetes *mellitus*) and socio-sanitary conditions (garbage collection).

Usually, the infection by *Mycobacterium tuberculosis* is linked to poverty, precarious conditions of housing in urban clusters (overcrowding), and to the reduction of social and sanitary resources, which explains why having garbage collected was a protection factor against TB latent infection^6^^,^ 10.

Studies conducted in the Northeast of Brazil on the distribution of TB cases found a close relation between the occurrence of the disease and areas of greater social vulnerability with the worst indications of quality of life, showing that the social problems of the population have a direct influence on the onset of the disease^11^.

On the other hand, our data found a protection relation between the presence of CKD and diabetes *mellitus*; in contrast to the literature, which points out that there is a moderately increased risk of latent infection by TB in patients with diabetes. This disease compromises the immune system and increases the general risk, up to three times, of the infection to evolve into active TB^12^^-^ 13. However, this same impairment of the immune response must also be one of the responsible elements for the lower response of the individual in the TST^12^^-^ 14.

Due to this dubiousness in the literature, the screening of TB latent infection in diabetic people must be combined with other risk factors and comorbidities for TB to better identify the high-risk groups and improve the tracking effectiveness. The American Thoracic Society recommends that the tuberculin skin test be performed in all patients with diabetes *mellitus*. In cases in which presence of a firm, hyperemic protuberance, larger than 10 mm, on the application spot, the administration of Isoniazid for 6 to 12 months is suggested^14^. Thus, based on the results of this study, the interpretation of the tuberculin skin test must consider the clinical-epidemiological context, in addition to other risk factor for latent TB.

In Brazil, the vaccination with the Calmette-Guérin *bacillus* is recommended at birth, favoring a protective factor against the development of TB, especially in the meningeal and disseminated forms of childhood and in false-positive results in the tuberculin skin test already in adult life^15^. In the present study, there was evidence of previous vaccination against TB (they reported having taken the vaccine and had the vaccine scar detectable) in 89% of the CKD patients. This suggests that the purified protein derivative had little interference with the TST result of these adults^16^, since only 8.5% of them had a reactor test when the expected would be around 25% to 30%. The recommendation manual for TB control in Brazil indicates that the TST might cause some interference with the reading and cutoff point to consider a person reactor, only in the first two years after its application in children who receive the vaccine in their first month of life^8^.

Thus, the results of this research are opportune and of great importance, as they highlight the prevalence of TB and can have an important association with previous comorbidities and socioeconomic conditions, highlighting the need for better tracking of latent TB among people with chronic conditions like the one herein studied. However, there is still lack of algorithms and/or protocols with this intention at the national or international level, which makes this task ineffective.

This research has as a limitation the fact that the collection of data on exposure and the outcome occurred simultaneously, a circumstance that makes it difficult to establish risks, as well as to understand the existing temporal relationship. Further studies, especially longitudinal, may provide subsidies to overcome this limitation. Another limitation refers to having been conducted in only one institution, which can limit data generalization.

## Conclusion

The prevalence of latent TB among patients with CKDon hemodialysis was not high. Among the predictive factors for latent TB, the presence of pre-existing comorbidity (diabetes) and a socio-sanitary condition (garbage collection) stand out. Therefore, it is necessary to be attentive to the possibility of developing active TB, with a generally extra-pulmonary clinical presentation or spread in these patients, since the diagnosis tests cannot detect all the reactors, and TB is associated with chronic disease and unhealthy environments, contributing to the high rates of morbidity and mortality of this population.

Considering that this is a highly prevalent disease on a world scale, focusing on latent TB among people with chronic diseases, like those with CKD, may increase the diagnosis capacity and lead the creation of algorithms and/or protocol with this purpose.
